# Implementing electronic patient reported outcomes in inflammatory bowel disease: patient participation, score reliability and validity

**DOI:** 10.1186/s12955-023-02087-0

**Published:** 2023-01-13

**Authors:** Daniel Deutscher, Clara Weil, Gabriel Chodick, Sveta Tsukinovsky, Keith Bodger, Matti Waterman, Revital Kariv

**Affiliations:** 1 Maccabitech Institute for Research & Innovation, Maccabi Healthcare Services, 4 Kaufmann St. Sharbat House, 8th Floor, 6801200 Tel Aviv, Israel; 2 Net Health Systems, Pittsburg, PA USA; 3 Sackler Faculty of Medicine, Tel Aviv University, Tel Aviv, Israel; 4 Takeda Pharmaceuticals, Petach Tikva, Israel; 5 Department of Health Data Science, Institute of Population Health, University of Liverpool, Liverpool, UK; 6 Digestive Diseases Unit, Aintree University Hospital NHS Trust, Liverpool, UK; 7 B. Rappaport Faculty of Medicine, The Technion – Israel Institute of Technology, Haifa, Israel; 8 Department of Gastroenterology, Rambam Health Care Campus, Haifa, Israel; 9 Health Division, Maccabi Healthcare Services, Tel-Aviv, Israel

**Keywords:** IBD control, Patient reported outcome measure, Patient participation, Reliability, Validity

## Abstract

**Background:**

Patient-reported outcome measures (PROMs) are recommended for assessing patient-centered outcomes in inflammatory bowel disease (IBD). The main aims were to assess the level of participation in an electronic PROM (ePROM) data collection system among patients with IBD, and evaluate reliability and validity of the resulting scores.

**Methods:**

Patients included in the IBD registry of Maccabi Healthcare Services, a state-mandated healthcare provider for over 2.6 million people in Israel, were invited to complete the IBD-Control measure and a general health item, with follow-up ePROMs at 3 and 6 months including a global rating of change item. Descriptive statistics were used to compare patient characteristics by participation rate, and assess survey completion time. Initial scores were assessed for internal consistency reliability using Cronbach's alpha. Test–retest reliability was assessed using the intraclass correlation coefficient from paired scores of patients identified as unchanged between the initial and first follow-up. Construct validity was assessed by the ability of IBD-control scores to discriminate between patient sub-groups in expected ways. Empirical validity was assessed using ePROM score correlations with laboratory markers of disease activity. Score coverage was also assessed.

**Results:**

A total of 13,588 patients were invited to participate [Mean age = 49 years (SD = 17); females = 51%]. Participation rate was 31.5%. Participants compared to non-participants were slightly older, were more likely to be female, to have a history of biologic treatment, to have higher socio-economic status, and to be more experienced in the usage of the digital patient portal. Median survey completion time was approximately 1:30 min. Internal consistency and test–retest reliability were 0.86 and 0.98, respectively. Scores discriminated between patient sub-groups in clinically expected ways, with expected correlations to laboratory markers of disease activity. A notable ceiling effect was observed (> 15%) for IBD-Control scores.

**Conclusions:**

Feasibility, reliability, and validity of the ePROM system was supported for measuring the level of perceived disease control in patients diagnosed with IBD in Israel. Additional research is needed to identify ways to increase patient participation, assess clinical implications of the identified measurement ceiling of the IBD-control, and evaluate the added value of the derived scores in support of clinical decision making.

## Background

Patient-reported outcome measures (PROMs) translate the patient’s experience into a measurable construct that can be used to monitor perceived health status over time [1, 2]. PROMs have been recommended for assessing patient-centered outcomes in Inflammatory bowel disease (IBD) combined with objective measures of inflammation [3, 4]. However, implementation of PROMs in routine practice is challenging, requiring patient compliance and integration of patients’ perception into clinical assessments and decision making processes. To maximize patient compliance and physician participation, reliable and valid short PROMs were developed [5], including the IBD-Control used in this study [6].

The IBD-Control, developed by Bodger et al. [6], is comprised of 13 items (questions) and a visual analogue scale (IBD-Control-VAS). Eight of the 13 items are used for scoring (IBD-Control-8). The IBD-Control was found to be reliable, valid against more complex health related quality of life tools including the UK version of the IBDQ [7] and the EuroQol (EQ-5D) [8], and sensitive for measuring overall disease control from the patient’s perspective [6]. The IBD-Control was recommended for use in pragmatic clinical trials [3], and as a single PROM included within a minimum standard set of patient-centered outcome measures for IBD [9]. Digital platforms have been suggested as appropriate means for electronic PROMs (ePROMs) data collection [10], offering data integration into electronic medical records with minimal burden, driving the aims of this study.

## Methods

### Aim

This study aimed to assess the implementation of a self-reported digital PROM data collection system among patients with IBD within a large nationwide state-mandated healthcare provider in Israel, Maccabi Healthcare Services (MHS), and test reliability and validity of the resulting scores.

### Design and setting

A prospective observational cohort study (longitudinal survey design).

### Participants and data collection period

Patients aged 18 or older who were registered in the MHS's IBD registry [11–13] were invited to participate during April 2019. A detailed description of the development and validation of the IBD registry algorithm has been published previously [11]. Briefly, the ascertainment of IBD cases utilizes three validated algorithms: (1) for identifying patients with a diagnosis of IBD; (2) for detecting the date of disease diagnosis, and (3) for identifying Crohn's Disease (CD) versus ulcerative colitis (UC) versus unclassified-IBD (IBD-U). The algorithms utilize two main criteria: (1) a combination of IBD-related ICD-9 codes when more than one code exists in the electronic health record; or (2) a combination of ICD-9 codes with at least three purchases of IBD-related medications with at least a 3-month interval from first to last purchase (sensitivity 89%, specificity 99%, positive predictive value [PPV] 92%, negative predictive value [NPV] 99%). IBD type was established according to the majority of CD/UC-specific codes out of the three most recent healthcare contacts, or the most recent contact when fewer than three were recorded (sensitivity 92%, specificity 97%, PPV 97%, NPV 92%). Only patients with a documented date of disease diagnosis were included. IBD-U type was identified according to a third algorithm, based on a specific code which exists for this condition in MHS [11–13]. No exclusion criteria were applied. Patients who completed an initial ePROM were invited to complete follow-up PROMs at 3 and 6 months.

### Outcome measures

The ePROMs administered included 3 domains: (1) The general health item from the Patient-Reported Outcomes Measurement Information System (PROMIS) global measure [14]; (2) The IBD-Control-8 and the IBD-Control-VAS [6]; and (3) at follow-up, a Global Rating of Change (GRoC) item with a 15-point scale for the degree of change (-7 to + 7), with zero representing no change [15].

### Survey administration process

Patients were invited to participate via a text message using the MHS patient portal messaging system, including a reminder after 3 working days, and thereafter, 2 additional reminders at one-week intervals. After successful identification on the secured patient portal, a landing page presented information about the study and the estimated completion time (2 min), inviting patients to complete the ePROM. Patients were informed that their survey data would not be shared with care providers, but would remain available to them, enabling self-tracking and sharing with their physician at their discretion. Four selections were available on the landing page: (1) participate, (2) postpone participation to a later time, (3) decline participation, or (4) decline stating they are not diagnosed with IBD. Selecting ‘participate’ was considered as agreement to participate in the study, and no other consent was required. After completion, a summary screen was presented including the IBD-Control-8 total score and score direction (higher scores = better IBD control). No other clinical interpretations or recommendations were provided. Available validated translations were obtained from the measure developers for the PROMIS global health PROM. The IBD-Control was translated into Hebrew, Russian, and Arabic by a professional translation team using validated methods [16].

### Analyses

#### Patient sample

Health and demographic baseline patient characteristics were summarized by IBD type (CD, UC, or IBD-U) using distribution or dispersion measures as appropriate. Variables were years since the patient was included in the IBD registry, age, sex, biologic treatment, and socioeconomic-status (SES). Biologic treatment was considered as a single surrogate marker for disease severity, categorized as a binary (yes/no) variable defined as having ever purchased at least one biologic/small molecule drug including: Vedolizumab, Infliximab, Adalimumab, Ustekinumab, Golimumab, Tofacitinib, or Certolizumab pegol. SES levels, built for commercial purposes by Points Location Intelligence, were defined by residential areas ranked from 1 (lowest) to 10, and categorized by tertiles into low (1–5), medium (6–7) and high (8–10), and correlated highly with SES measured by the Israel Central Bureau of Statistics [17]. *P*-values for statistically significant differences were estimated using Chi-square tests for comparisons of categorical data and analysis of variance for comparisons of continuous data. However, due to the large cohort, statistically significant differences need to be interpreted with caution.

#### Participation rate

Participation rate was operationally defined as the percentage of patients reaching the landing page, stratified by full or partial completion, or by reasons for declining to participate. Participation was tested separately for the initial survey and for the two follow-up surveys, and by patient subgroups offering insights on differences in patient attributes by participation. Variables included age groups, sex, IBD type (CD, UC, or IBD-U), use of biologic treatment, SES, and digital platform usage during the past 12 months, including no use, or one of four digital usage levels defined by quartiles of digital log counts. To assess the potential for patient participation bias, an effect size was calculated as the standardized difference in participation rates between participants and non-participants for the variables listed above [18]. An effect size below 0.2 was considered as representing a non-meaningful difference [19]. Additionally, a multivariable logistic regression was used to estimate the likelihood of participating while accounting for all factors above.

#### PROM scores and completion time

PROM scores were assessed by survey type (initial or follow-up) and domain (general health and IBD-Control). Score values (mean, SD, median), as well as survey completion time, were also assessed. Survey completion time was assessed for all complete surveys with a completion time between 30 s and 1 h, assuming times outside these limits represented outliers, or surveys completed over multiple instances.

#### Reliability of point estimates and change scores

Internal consistency reliability for the IBD-Control-8 was assessed using initial scores with Cronbach's alpha. The standard error of measurement (SEM) was calculated by multiplying the standard deviation by the squared-root of 1-(minus) the reliability estimate, in this case Cronbach's alpha [20]. Different confidence intervals (CIs) were computed including the 68% CI, which is equivalent to 1 SEM, and 80%, 90%, and 95% CIs. Reliability of change scores was assessed using the minimal detectable change (MDC), reflecting the minimal amount of change that is beyond measurement error, at different levels of confidence. Since change involves at least two measured points, reliability-based estimates of MDC were calculated by multiplying the SEM of the difference (SEM_difference_ = SEM * square-root of 2) by the appropriate Z-value [20]. Test–retest reliability was assessed using the intraclass correlation coefficient (ICC) from pairs of IBD-Control-8 scores (initial and first follow-up) of patients identified as unchanged between these two measurement points [21]. Unchanged patients were defined as those that had a GRoC score at their first follow-up ePROM of − 2 to + 2, reflecting change that is less than minimally important to patients [22].

#### Validity

*Empirical validity* was assessed by testing associations between the IBD-Control-8 scores and two related scores including the IBD-Control-VAS and general health scores. Since all ePROM assessed have the same direction (higher = better), we expected positive moderate correlations or higher, which in the context assessed here, were determined to be above 0.3 [23]. We also expected a higher correlation within domain (IBD-Control-8 and IBD-Control-VAS), compared to correlations between each of these to the general health domain. Additionally, correlations of IBD-Control-8 scores with laboratory markers of inflammation and disease activity, including albumin, hemoglobin, and calprotectin, were tested at 15 days before or after the date of the ePROM. Calprotectin performance may differ between UC and CD; therefore, we analyzed these groups separately [24]. Low significant correlations in a clinically logical direction were expected. Since we were not aware of known differences between CD and UC regarding correlations of PROMs and laboratory markers, we considered these analyses exploratory rather than hypothesis driven. To account for ordinal level ePROM scores, Spearman’s rank correlations were used.

*Discriminant validity* was assessed by testing if IBD scores discriminated between patient groups in expected clinical patterns. Although existing evidence on associations between self-assessed IBD disease control and patient demographic and health characteristics are unclear, given previous reports, we expected higher IBD-Control for patients who were older, were males, were diagnosed with UC, and had never purchased biological medications (lower severity) [25–28]. Group differences were tested for the initial IBD-scores using ANOVA.

*Score coverage* was used to assess floor and ceiling effects. We defined maximally acceptable floor and ceiling effects as 15% of sample scores in the minimum or maximum score of the IBD-Control-8 and the general health question, and the minimum or maximum range of 0–5 and 95–100, respectively, for the IBD-Control-VAS [29, 30].

All analyses were performed using IBM SPSS, version 25.0.0.1 [31] and Stata version 14 [32].

## Results

### Patient sample

A total of 13,588 patients were invited to participate [Mean age (SD) = 48.9 (16.6); females = 50.5%; Table 1]. Compared to patients diagnosed with UC, those diagnosed with CD were on average 5 years younger, less likely to be female, and more likely to have a history of biologic and small molecule treatment use indicative of higher levels of disease severity. The distributions of SES levels were similar between IBD types. For patients who responded to the initial survey (n = 4280), the majority selected to respond in Hebrew (93.6%), followed by 3.4%, 2.6%, and 0.4% for patients responding in Russian, English, and Arabic, respectively.

**Table 1:** Patient sample by IBD type

Patient characteristics	CD n = 6917	UC n = 6118	Unclassified n = 553	Total N = 13,588	*P*
Years in IBD registry^a^ Median (Min–Max) 25th; 75th percentiles	9 (0–19) 4; 15	11 (0–19) 5; 17	8 (0–19) 4; 13	10 (0–19) 5; 15	< 0.001
Age: Mean (SD)Median (Min–Max) Median (Min–Max) 25th;75th percentiles	45.9 (15.9) 44.7 (19–100) 33.0; 56.4	52.1 (16.7) 51.2 (19–101) 39.4; 64.3	51.5 (18.0) 49.8 (19–102) 37.0; 64.5	48.9 (16.6) 47.8 (19–102) 35.7; 60.7	< 0.001
Age groups:					< 0.001
18–45	3512 (50.8)	2155 (35.2)	206 (37.3)	5873 (43.2)	
> 45–65	2422 (35.0)	2493 (40.8)	212 (38.3)	5127 (37.7)	
Over 65	983 (14.2)	1470 (24.0)	135 (24.4)	2588 (19.1)	
Sex:					< 0.001
Female	3381 (48.9)	3167 (51.8)	315 (57.0)	6863 (50.5)	
Male	3536 (51.1)	2951 (48.2)	238 (43.0)	6725 (49.5)	
Biologic treatment^b^					< 0.001
Yes	2538 (36.7)	742 (12.1)	88 (15.9)	3368 (24.8)	
No (never)	4379 (63.3)	5376 (87.9)	465 (84.1)	10,220 (75.2)	
SES					0.769
1 to 5 (low)	1685 (24.4)	1443 (23.6)	132 (23.9)	3260 (24.0)	
6 to 7 (moderate)	2610 (37.7)	2355 (38.5)	211 (38.2)	5176 (38.2)	
8 to 10 (high)	2606 (37.7)	2305 (37.7)	207 (37.4)	5118 (37.8)	
Missing	16 (0.2)	15 (0.2)	3 (0.5)	34 (0.9)	

Values are n (column %) unless noted otherwise. *P*-values for statistically significant differences were estimated using Chi-square tests for comparisons of categorical data and analysis of variance for comparisons of continuous data

^a^Year of inclusion in the IBD registry at the start of 2019. Zero represents less than 1 year within the registry

^b^Biologic and small molecules treatment was defined as having purchased at least one biologic medication including: Vedolizumab, Infliximab, Adalimumab, Ustekinumab, Golimumab, Tofacitinib, or Certolizumab

*CD* Crohn's disease; *UC* Ulcerative Colitis; *IQR* inter quartile range; *SD* standard deviation; *SES* socioeconomic status

### Participation rate

**Fig. 1:**
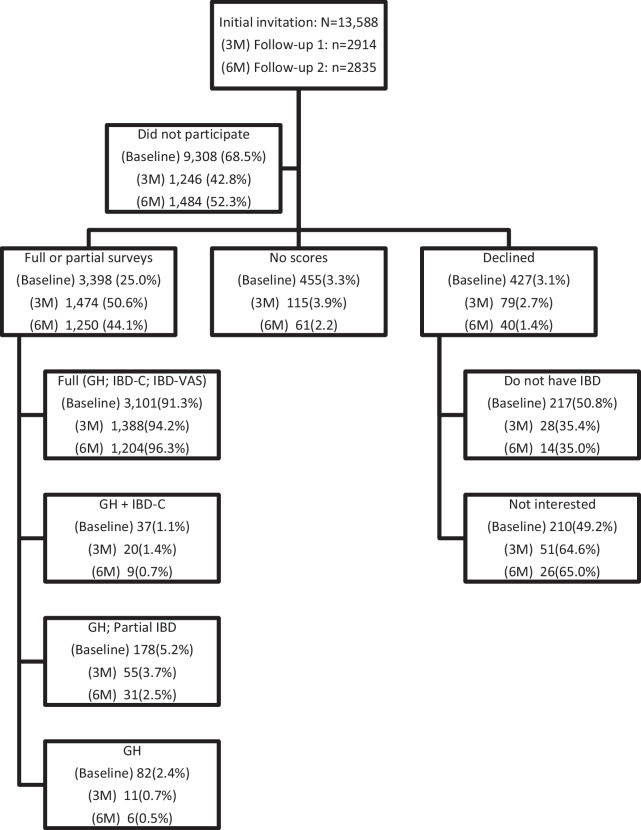
Participation in the initial and two follow-up surveys. Participation rate was operationally defined as the percentage of patients selecting the web link on the invitation text message and reaching the landing page after a successful identification. Percentages are from the level above for the corresponding survey number. For example, at baseline, 68.5% of patients did not participate, with 25.0% (3398/13,588) having full or partial survey completion, 3.3% reached the landing page but did not complete any survey item therefor had no scores, and 3.1% declined participation selecting reasons of not having IBD or not interested to participate, summing up to an overall participation rate of 31.5%. For those with full or partial survey completion, the distribution of score combination is shown for those with all three scores (global health, IBD-control, and IBD-VAS), or partial score combinations. *3 M* first follow-up at three months; *6 M* second follow-up at six months; *IBD* Inflammatory bowed disease; *GH* General health score from the Patient-Reported Outcomes Measurement Information System (PROMIS) general health item; *IBD-C* IBD-Control-8 scores; *IBD-VAS* IBD visual analog scale scores

Participation rates for the initial survey by age, sex, IBD type, IBD severity, SES levels and digital platform usage are presented in Table 2. The overall participation rate was 31.5%. All standardized differences were < 0.2, except for the 'low' SES category and all except 'moderate' digital usage categories. Results from the multivariable logistic model indicated that patients were more likely to participate if they were older, had not received biologic treatment, had a moderate (compared to low) SES level, and had moderate or higher levels of digital usage. A more detailed illustration of participation in the initial survey (baseline) and the two follow-up surveys are illustrated in the Fig. 1. Overall, participation rates for the first and second follow-up surveys from those who responded to the previous survey administration were 57% and 48%, respectively. The percentage of patients with no scores ranged from 2.2 to 3.9%, and the percentage of patients who declined participation decreased between the initial and the 2nd follow-up survey from 3.1% to 1.4%.

**Table 2:** Patient characteristics by participation in the initial survey

Patient characteristics	Participated n = 4280	Did not participate n = 9308	Standardized difference^b^	Odds ratio^c^ (95% CI)
Age: Mean (SD) Median (Min to Max)	49.7 (15.1) 49.1 (19–95)	48.5 (17.3) 47.7 (19–102)	0.07	NA
Age groups				
18–45	1648 (38.5)	4225 (45.4)	0.14	REF
> 45–65	1866 (43.6)	3261 (35.0)	0.18	1.9 (1.7–2.1)
Over 65	766 (17.9)	1822 (19.6)	0.04	1.6 (1.5–1.8)
Sex				
Female	2304 (53.8)	4559 (49.0)	0.10	REF
Male	1976 (46.2)	4749 (51.0)		1.0 (0.9–1.1)
IBD type				
CD	2182 (51.0)	4735 (50.9)	< 0.01	REF
UC	1938 (45.3)	4180 (44.9)	0.01	1.1 (1.0–1.1)
Unspecified	160 (3.7)	393 (4.2)	0.03	0.9 (0.7–1.1)
Biologic treatment ^a^				
Yes	1136 (26.5)	2232 (24.0)	0.06	REF
No (bio-naïve)	3144 (73.5)	7076 (76.0)		1.1 (1.0–1.2)
SES				
1 to 5 (low)	769 (18.0)	2491 (26.8)	0.21	REF
6 to 7 (moderate)	1708 (39.9)	3468 (37.3)	0.05	1.2 (1.1–1.3)
8 to 10 (high)	1792 (41.9)	3326 (35.7)	0.13	1.1 (1.0–1.2)
Missing	11(0.3)	23 (0.2)	0.02	1.0 (0.5–2.1)
Digital usage count (past year)				
None	38 (0.9)	1450 (15.6)	0.55	0.1 (0.1–0.2)
Low (1 to 19)	519 (12.1)	2573 (27.6)	0.40	REF
Moderate (20 to 46)	1004 (23.5)	1926 (20.7)	0.07	2.7 (2.4–3.0)
High (47 to 94)	1218 (28.5)	1823 (19.6)	0.21	3.6 (3.2–4.1)
Very high (95 or more)	1501 (35.1)	1536 (16.5)	0.43	5.8 (5.1–6.6)

Values are n (column %) unless noted otherwise

Total percentages may range between 99.9–100.1 due to rounding

^a^Biologic treatment was defined as having purchased at least one biologic medication including: Vedolizumab, Infliximab, Adalimumab, Ustekinumab, Golimumab, Tofacitinib, or Certolizumab

^b^The absolute standardized differences was calculated as described by Austin [18] Standardized differences below 0.2 were considered non-meaningful

*CI* confidence level, *NA* not applicable, *REF* reference group, *SES* Socioeconomic status, *CD* Crohn's disease; *UC* Ulcerative Colitis

^c^Odds ratios are mutually adjusted for all variables in the table, estimated from a multivariable logistic regression that modeled the likelihood of participating compared to the reference group

### PROM scores and completion time

Score counts, summary values, and overall completion time by survey type (initial or follow-up) are presented in Table 3. From 6122 surveys collected, 5759 had complete IBD-Control-8 scores. Median survey completion time for initial, first follow-up, and second follow-up surveys were all approximately 1:30 min.

**Table 3:** Scores by survey and domain and survey completion time

Domain	Initial survey	Follow-up 1	Follow-up 2
General Health			
Counts	3398	1474	1250
Median (25th; 75th percentiles)	3(3; 4)	3(2; 4)	3(3; 4)
Min–max	1–5	1–5	1–5
IBD-Control-8			
Counts	3138	1408	1213
Median (25th; 75th percentiles)	13(8; 15)	13(8; 16)	14(9; 16)
Min–max	0–16	0–16	0–16
IBD-Control VAS			
Counts	3101	1388	1204
Median (25th; 75th percentiles)	74(50; 90)	72(49; 89)	76(51.5; 90)
Min–max	0–100	0–100	0–100
Total survey completion time (minutes)^a^			
Counts^b^	3047	1360	1175
Median (25th; 75th percentiles)	1:28(1:11; 1:56)	1:34(1:15; 2:05)	1:35(1:15; 2:08)

*IBD* inflammatory bowel disease; *VAS* visual analog scale

^a^Completion time reflect to total time needed to complete the full survey

^b^Counts include surveys with a completion time between 30 s and 1 h, assuming times outside these limits represented outliers, or surveys completed over multiple instances

### Reliability of point estimates and change scores

Internal consistency reliability for the IBD-Control-8 was 0.86. The SEM was 1.7 points. Reliability of point estimates at 80%, 90%, and 95% levels of confidence were 2.2, 2.8, and 3.4 points, respectively. MDC at 68%, 80%, 90%, and 95% levels of confidence were 2.4, 3.1, 4.0, and 4.8 points, respectively. IBD-Control-8 test–retest reliability (ICC) using scores from 918 patients identified as unchanged was 0.968 (95%CI = 0.963–0.972).

### Validity

#### Empirical validity

Bi-variate correlation coefficients between IBD-Control-8 scores, IBD-Control-VAS scores, and general health scores, were all above 0.6. As hypothesized, all correlations were positive, with a higher correlation found between IBD-Control-8 and IBD-Control-VAS (Spearman's rank correlation = 0.77) compared to correlations between each of these to the general health scores ranging from 0.63 to 0.64. All correlation coefficients were significant (*P* < 0.001). IBD-Control-8 correlations with laboratory markers of inflammation and disease activity were in the expected directions (Table 4). Correlations were overall low but significant for most tests, with the highest correlation observed between IBD-Control-8 scores and fecal calprotectin for patients with UC.

**Table 4:** IBD-Control-8 score correlations with laboratory markers

	CD	UC
Albumin	.192**(375)	.187**(232)
Calprotectin	− .106(143)	− .314*(41)
Hemoglobin	.139**(530)	.213**(352)

Values are Spearman's rank correlation coefficients (n)

Time between the date of the ePROM and the laboratory test =  + / − 15 days

^*^P < 0.05; **P < 0.01

*CD* Crohn's disease; *UC* Ulcerative Colitis

#### Discriminant validity

IBD scores discriminated between patient groups in expected clinical patterns (Table 5), with higher IBD-Control found for patients who were older, were males, were diagnosed with UC, and had never purchased biological medications.

**Table 5:** Discriminant validity

Patient characteristic		Model (ANOVA)			Marginal means (IBD-Control-8)		
Variable	Groups	N	%	F(df) Prob > F	b	95% CI	
Age	18–45	1267	40.4%	15.5(2) *P* < 0.001	10.6	10.3	10.8
	45–65	1368	43.6%		11.2	11.0	11.5
	65 to max	503	16.0%		11.9	11.5	12.3
Gender	Male	1443	46.0%	29.77(1) *P* < 0.001	11.6	11.3	11.8
	Female	1695	54.0%		10.7	10.4	10.9
IBD type	CD	1626	51.8%	19.8(2) *P* < 0.001	10.6	10.4	10.8
	UC	1427	45.5%		11.6	11.4	11.9
	Unclassified	85	2.7%		10.8	9.8	11.8
*Biologic treatment	No	2225	70.9%	163.2(1) *P* < 0.001	11.7	11.6	11.9
	Yes	913	29.1%		9.4	9.1	9.7

Group differences were tested for the initial IBD-Control scores (N = 3138)

Marginal means are for IBD-Control-8 scores (0–16 scale)

^*^Biologic treatment was defined as having purchased at least one biologic medication including: Vedolizumab, Infliximab, Adalimumab, Ustekinumab, Golimumab, Tofacitinib, or Certolizumab

*b* beta coefficient, *df* degrees of freedom, *CD* Crohn's disease; *UC* Ulcerative Colitis

#### Score coverage

Floor and ceiling effects for IBD-Control-8 scores, IBD-Control-VAS scores, and general health scores, for the initial and the two follow-up surveys, are presented in Table 6. Floor effects were all below 15%, with negligible floor effects for the IBD-Control-8 and IBD-Control-VAS scores (< 2%). IBD-Control-8 and IBD-Control-VAS scores had notable ceiling effects ranging from 17 to 30%.

**Table 6:** Score coverage

Floor and ceiling effects (%)			
	Initial survey	Follow-up 1	Follow-up 2
General Health (min/max)	6.9/14.0	8.4/11.1	4.6/15.6
IBD-Control-8 (min/max)	1.4/22.7	1.8/25.1	1.2/30.0
IBD-Control-VAS (0–5/95–100)	1.7/19.9	1.8/16.6	0.9/19.3

Values are in percent (Floor/Ceiling)

Floor and ceiling effects were defined as the minimum or maximum score of the IBD-Control-8 scores (0 and 16) and the general health scores (1 or 5), respectively, and the minimum or maximum range of 0–5 and 95–100, respectively, for the IBD-Control-VAS

## Discussion

We describe in this report the feasibility and measurement properties of an ePROM platform among IBD patients in a real-world setting. The relatively high response rate along with extremely short completion time, attest to its feasibility and potential for implementation in routine clinical practice and research initiatives. Essential psychometric properties of reliability and validity of the generated IBD-Control-8 scores were supported, increasing confidence in their precision and potential capacity to serve as a viable and valid source of information for patients and clinicians. These results should be interpreted within the context of the population tested, including mostly Hebrew speaking IBD patients in Israel.

Participation rate was 31.5% for the initial survey, increasing up to 48–57% for follow-up surveys. Over 90% of patients who started the survey completed the full set of scores including the general health item, IBD-Control-8, and IBD-Control-VAS. These participation rates are encouraging given that the framework of this study did not include any direct patient-clinician interaction related to the ePROM data collection process. Studies assessing ePROM participation rates, usually within a clinical trial or before scheduled clinical visits, reported participation rates ranging from 33 to 74% [33, 34], suggesting a potential for improved participation rates when ePROMs are implemented within a clinical setting. Recent evidence exists of improved healthcare management, physician–patient communication, and symptom detection following routine clinical use of PROMs data [35]. This may encourage physicians to engage their patients in routine PROM completion to enable self-monitoring and assist clinical decision making. The feasibility of an ePROM platform as used for this study is supported by previous findings [36], suggesting this approach could be scalable for wide range of portals and apps among IBD patients in other healthcare systems. However, the lower participation rates observed among patients with lower SES levels, or those less experienced with the use of digital portals, suggests a potential barrier of ePROMs implementation within populations that are often at risk of having lower health status. This emphasizes the need for ePROM implementation studies to assess their usability in different patient populations.

A key element to successful implementation of PROMs data collection is low survey administration burden. Survey completion time in our study was roughly 1:30 min and was similar to the timing reported by Bodger et al. [6] 1:15 min. We consider these results to not pose a barrier to patients when considering participating in ePROMs data collection. Older age has also been reported as an additional barrier to digital PROM participation [37]. Our results did not identify important differences in mean age by participation (standardized difference = 0.07). Also, standardized differences in rates of patients by age groups between participants and non-participants were all < 0.2, suggesting age was not a critical barrier for ePROM completion, as suggested previously [36].

The reliability estimates provided may help clinicians assess measurement error associated with a point estimate or a change score. For example, reliability estimates show that there is a 90% confidence that the true patient score falls within + / − 2.8 IBD-Control-8 points on the 0–16 scale. As an example, if used in conjunction with a threshold value of 13 that has been suggested to represent a state of quiescent (high level of IBD control) [6], only a perfect score of 16 (13 + 2.8) would provide this level of confidence that the patient has in fact been quiescent. Additionally, results suggest 4 or 5 change points are needed to represent true change at a 90% or 95% confidence, respectively.

Correlations between ePROM scores with several laboratory tests that may indicate disease activity or severity were low and in the expected directions, supporting the validity of the IBD-Control-8 scores. Interestingly, although correlations of albumin and hemoglobin with IBD-Control-8 were similar between CD and UC, calprotectin correlations were higher for UC compared to CD. Overall, this is not surprising as fecal calprotectin correlates better with the level of inflammation in UC than in CD [38]. Correlations between objective markers of inflammation and disease activity, and subjective measures of disease control, are not expected to be high as they assess two related but distinct constructs. Patient-perceived global control of disease in IBD may reflect both inflammatory and non-inflammatory manifestations of disease, co-existing functional symptoms and impacts of medication. Therefore, these results need to be interpreted with caution. The key use-case for the PROM is to serve as an additional marker of health status. Those reporting sub-optimal scores may have non-inflammatory drivers of their self-assessed IBD control rating, but they still have health needs to be addressed. Thus, a formal clinical assessment combined with objective tests is needed to distinguish between those with active inflammation and those with other reasons for sub-optimal PROM scores.

The main strength of this study was the large number of patients selected from a generalizable IBD registry. Also, the use of an easily accessible mobile-based digital platform to collect patient self-reported outcomes offers a novel method to improve patient centered care.

However, this study has some notable limitations. Initial surveys were completed fully or partially by 25% to 31% of all of the target patient population, respectively. Although these participation rates could be considered high given that ePROMs were not part of a clinical interaction, they also pose a potential patient participation bias that might distort the assessment of the true patient population of interest. This bias may lie in the survey's electronic administration mode, a limitation supported by our finding of higher likelihood to participate for those more experienced with overall digital usage. This result highlights the need for future studies assessing the impact of a patient's 'digital profile' on ePROM feasibility. An important strength of the MHS setting is that it harbors full demographic and health data on both responders and non-responders, offering an excellent opportunity to study the potential of response bias. Some study patients were classified as ‘IBD-U’ or indeterminate colitis [39]. Currently, there is a lack of data on its epidemiology, clinical course, reclassification trends, and treatment responses. Using PROM data may help better understand these patients’ characteristics from a patient-centered perspective. Finally, score coverage results revealed a notable ceiling effect of IBD-Control-8 and IBD-Control-VAS scores. Additional studies are needed to assess whether the measured ceiling effect reflects a true positive state of IBD-control, or a psychometric limitation.

## Conclusion

The ePROM platform assessed was found feasible and suitable for clinical integration and research initiatives for patients with IBD in Israel, providing reliable and valid measures of the level of perceived disease control. This allows for an integration of ePROMs data within the electronic medical record, offering clinicians an improved ability to monitor levels of IBD control from the patient’s perspective.

## Data Availability

The datasets used and/or analyzed during the current study are available from the corresponding author on reasonable request.

## Abbreviations

CD: Crohn’s disease
CI: Confidence interval
ePROM: Electronic patient-reported outcome measures
GRoC: Global rating of change
IBD: Inflammatory bowel disease
IBD-U: Unclassified IBD
ICC: Intraclass correlation coefficient
MHS: Maccabi Healthcare Services
MDC: Minimal detectable change
PROMs: Patient-reported outcome measures
SD: Standard deviation
SEM: Standard error of measurement
SES: Socioeconomic-status
UC: Ulcerative colitis
VAS: Visual analogue scale
